# Turning Up the Heat: Ultrafast Hot Carrier Extraction in FAPbBr_3_

**DOI:** 10.1021/acscentsci.3c01571

**Published:** 2024-01-10

**Authors:** Sarah Wieghold, Colette M. Sullivan, Lea Nienhaus

**Affiliations:** † Advanced Photon Source, Argonne National Laboratory, Lemont, Illinois 60439, United States; ‡ Florida State University, Department of Chemistry, Tallahassee, Florida 32306, United States

A detailed understanding of the structural and electronic properties of materials is critical when designing superior materials for photovoltaic applications. In this issue of *ACS Central Science*, Omar F. Mohammed and co-workers explore the carrier dynamics at the interface between a halide perovskite and an organic electron acceptor to understand injection dynamics of hot and relaxed charge carriers.^1^

Organic–inorganic hybrid perovskites have attracted significant interest over the past decade as new alternative absorber materials for photovoltaics (PVs) due to their exceptional optoelectronic properties. However, despite reaching record efficiencies above 25%, the grand challenge for organic–inorganic hybrid perovskites is still related to their (in)stability and long-term efficiency, which effectively decrease PV performance over time. To overcome this hurdle, one promising approach is to fully characterize and track carrier dynamics across interfaces of the layered PV structure to understand the fundamental carrier recombination and extraction processes at the atomic and molecular levels.^2^ In particular, a deeper understanding of the physical processes, i.e., specifically the electron and hole injection processes at the interfaces of organic–inorganic systems, can help in designing superior materials for high-performance photovoltaic devices.

To unravel the charge extraction dynamics, transient absorption spectroscopy in the visible^3,4^ and near-infrared^5^ spectral region has commonly been employed in conjunction with photoluminescence lifetime experiments.^6^ However, these methods cannot fully represent the underlying picture of charge distribution and the molecular structural changes upon excitation. By contrast, mid-IR spectroscopy is a powerful technique that can reveal the hidden processes of charge carrier transfer and provide a deeper understanding of these complex dynamics at the molecular level.

**Figure 1:**
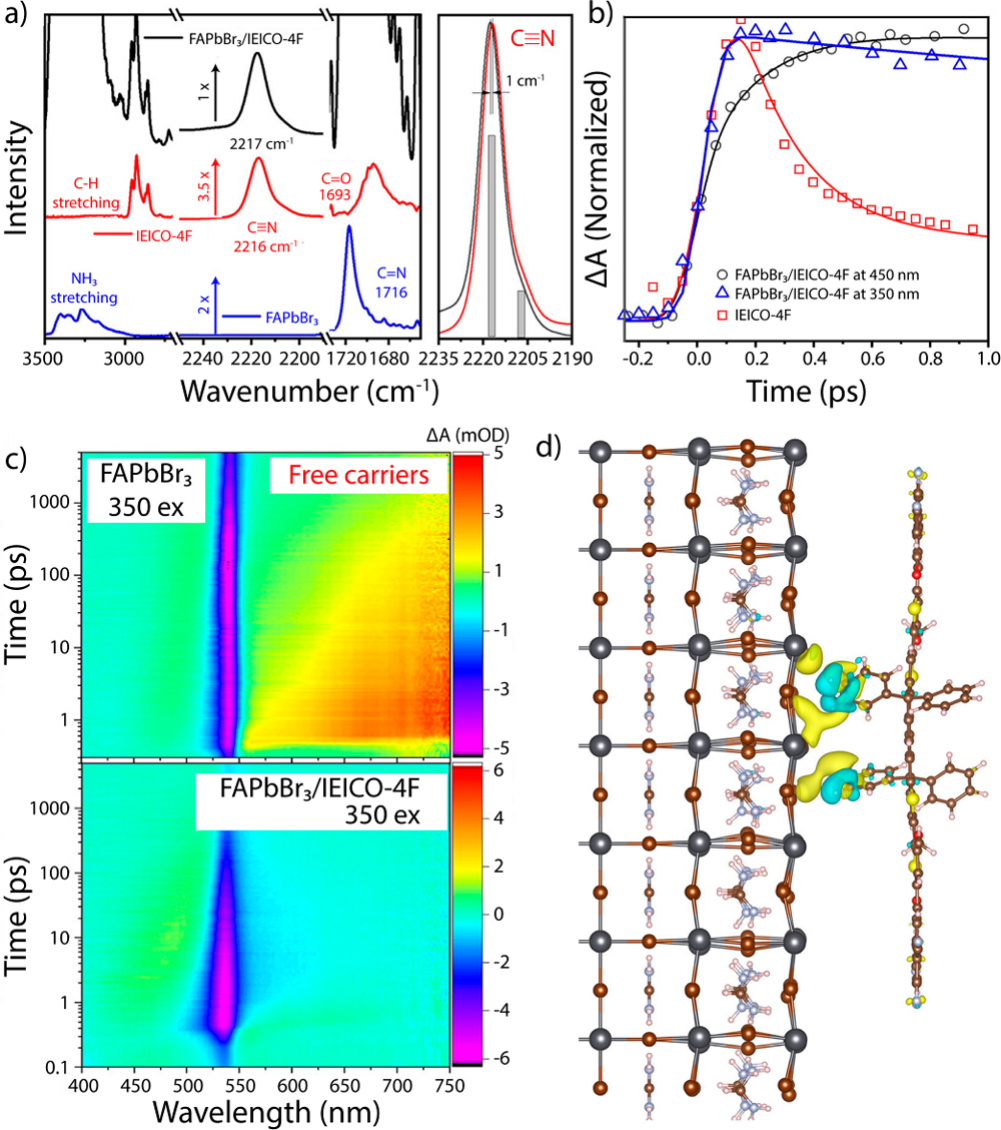
a) (Left) Steady-state Fourier transform infrared spectroscopy of the FAPbBr_3_/IEICO-4F (black), IEICO-4F (red), and FAPbBr_3_ (blue) films. (Right) ν(C≡N) vibrational mode of IEICO-4F. b) Kinetic traces of the mid-IR ν(C≡N) stretching mode at 2276 cm^–1^ for IEICO-4F (red squares) under 350 nm excitation and the FAPbBr_3_/IEICO-4F bilayers excited at 450 nm (black circles) and 350 nm (blue triangles). c) fs-TA spectroscopy maps for the FAPbBr_3_ (top) and FAPbBr_3_/IEICO-4F bilayer (bottom) under 350 nm excitation. d) DFT-generated charge density plot upon adsorption of IEICO-4F onto the FAPbBr_3_ [100] surface. Reproduced with permission from ref (1). Copyright 2023 American Chemical Society.

Here, electron injection is tracked at the interface between the formamidinium lead bromine (FAPbBr_3_) thin film as the electron donor and IEICO-4F, the electron acceptor. Figure 1a highlights the vibrational modes of interest within the system where the prominent ν(C≡N) vibrational mode at 2217 cm^–1^ for both IEICO-4F and the donor/acceptor bilayer serves as a point of reference for monitoring electron injection. Femtosecond (fs) mid-IR spectroscopy^7^ is used to investigate the characteristic vibrational modes of the acceptor and perovskite donor.

Mohammed and co-workers showed that the symmetrical and asymmetrical CN vibrational modes of the anionic IEICO-4F are involved in the ultrafast electron transfer process between the donor/perovskite and the acceptor. In particular, by tracking the CN vibrational modes via fs mid-IR spectroscopy, changes in the spectral and dynamic range between the neutral and anionic IEICO-4F were identified. Here, Figure 1b highlights the hot carrier extraction via tracking kinetic changes in the ν(C≡N) vibrational mode. Under 350 nm excitation (blue triangles), the FAPbBr_3_/IEICO-4F bilayer rises <1 ps, signifying a faster formation of the anionic IEICO-4F species compared to the 450 nm excitation (black circles). In addition, for the FAPbBr_3_ absorber alone, the generated hot carriers are transitioned to free carriers within 150 ps (Figure 1c, top). However, within the donor/acceptor system, these carriers are extracted from the perovskite into the electron acceptor within a matter of femtoseconds (Figure 1c, bottom).

Density function theory (DFT) calculations on the structure of IEICO-4F functional groups and the perovskite lattice suggest that the alkyl-substituted phenyl ring serves as the electron injection site. Visualizing the interface with charge density plots as shown in Figure 1d also provides valuable information regarding changes to the surface. Here, the authors found through DFT that no major deformations form far away from the interface. In addition, the DFT results show that the change from neutral to anionic within the electron acceptor causes a shift in the electron density distribution, resulting in a weakened CN vibrational mode able to be tracked via mid-IR spectroscopy. Thus, the present findings provide an enhanced understanding of the structural and electronic properties of these materials, along with useful insights for the development of new perovskite-based materials that are more efficient in light energy conversion.

In summary, the authors investigated carrier extraction at the FAPbBr_3_/IEICO-4F interface using fs mid-IR spectroscopy. Unraveling the ultrafast and time-resolved dynamics of this donor/acceptor system indicates that hot carriers are generated within the perovskite on an ultrafast time scale and are converted to free carriers within 150 fs, while on the other hand, in the presence of the IEICO-4F electron acceptor, the carriers are transferred on a femtosecond time scale. Of note is that the results indicate that the additional thermal energy of these hot carriers increases their extraction rate to the electron acceptor in comparison to thermalized carriers. By combining both fs mid-IR spectroscopy and DFT, Mohammed and co-workers were able to extract a detailed view of the FAPbBr_3_/IEICO-4F interface. Combining these techniques provides a powerful tool for elucidating interfacial structural changes and ultrafast charge extractions for donor/acceptor systems which can provide valuable insight into current PV technologies. It will also inform the design of material systems with tailored properties and the development of efficient optoelectronic devices in the future.
